# The epidemiology and characteristics of patients with diabetes with or without NASH: a systematic review

**DOI:** 10.4314/ahs.v23i2.59

**Published:** 2023-06

**Authors:** Khaled K Aldossari

**Affiliations:** Family & Community Medicine Department, College of Medicine, Prince Sattam Bin Abdulaziz University, Al-Kharj,11942, Saudi Arabia

**Keywords:** NAFLD, NASH, non-alcoholic steatohepatitis, diabetes mellitus, fatty liver, liver disease, systematic review

## Abstract

**Introduction:**

NASH or “Non-alcoholic Steatohepatitis” is related to non-alcoholic fatty liver disease (NAFLD). The simultaneous occurrence of NASH and type 2 diabetes is common. However, only a limited number of studies have focused on the characteristics of patients with diabetes with or without NASH.

**Objectives:**

This systematic review summarises epidemiological evidence related to the prevalence and characteristics of NASH in patients with diabetes.

**Methods:**

Different electronic databases PubMed, Scopus, and Google Scholar were searched for the published articles. Original studies conducted in patients with diabetes published in English were included in this review

**Results:**

Thirteen studies were eligible for inclusion in this review. In patients with diabetes, increased BMI, overweight/obesity, increased HbA1c, increased serum cholesterol, and elevated liver enzyme levels have been strongly linked with NASH. Other significant characteristics include increasing age, being female, race(white), low HDL, metformin use, increased ferritin, and increased albumin levels. The prevalence of NASH ranged from 12% to 93.8%, the highest percentage was found in studies in Romania (87.1), and lowest in studies in India (12.5).

**Conclusion:**

The incidence of NASH increases with age. Gender differences as a risk factor for NASH still need to be fully understood. This systematic review provides insight and strong indication to all patients with diabetes to visit hepatologists and screen for fatty liver disease. If steatosis is found on baseline ultrasound, a liver biopsy should be performed for timely management. At present, no NAFLD/NASH-specific medication on the market helps in treating the disease. New development of the drugs and ongoing research is important for the cure and treatment of NASH, with specific attention being provided to involve populations at high-risk.

## Introduction

Non-alcoholic Steatohepatitis (NASH) is a common, often “silent” progressive disease of the liver, which is primarily associated with either diabetes or obesity 1. NASH is a subset of non-alcoholic fatty liver disease (NAFLD) that requires effective treatment 2. This condition is similar to alcoholic liver disease, but it is usually found in individuals who consume either no alcohol or drink little alcohol. The key features of NASH are fat in the liver, accompanied by damage and inflammation. The majority of individuals with NASH usually feel well but are generally not mindful about their problems, leading to liver problems. However, NASH can be severe and may lead to cirrhosis, in which the liver becomes scarred or injured permanently or is incapable of working 3. The prevalence of NAFLD among the general population has increased from 15% to 25% in five years (2005-2010), whereas the prevalence of NASH has doubled from 33% to 59.1% in recent studies 1. Due to the growing prevalence of NASH, it is considered the second most common cause of liver transplant after chronic hepatitis C in the USA 4. Determination of the true NASH prevalence is usually complicated; liver biopsy provides the only definitive diagnosis of NAFLD/NASH. The cost or invasiveness of a liver biopsy obscures its use as a diagnostic tool for the general population 5. Blood tests are used to check liver enzymes, which are high when an individual suffers from NAFLD. After ruling out other liver diseases with an additional test, a diagnosis of NAFLD was confirmed with liver ultrasound. Liver tissue samples can often be taken for biopsy to distinguish NASH from NAFLD 6. Nevertheless, elevated liver enzymes, computed tomography, or ultrasound can be used as surrogate NASH markers. Histological features of NAFLD/NASH are usually similar to those observed in liver disease in the event of excessive alcohol intake.

Consequently, the diagnosis of NAFLD is clinicopathological, and an excess of alcohol intake (>20 g/day) may impede NAFLD diagnosis. NAFLD also includes a spectrum of pathological conditions, such as cirrhosis (scarring), non-alcoholic steatohepatitis (NASH) (inflammation), or simple steatosis. As the severity of NAFLD increases, so does morbidity, in addition to advanced fibrosis, which is a risk factor for progression to liver failure 5.

In addition to many clinical trials, no effective treatment for NASH has been reported 4. It has been indicated that it should focus on five pillars: intervention on lifestyles, pharmacological treatment of liver disease, dyslipidemia or hyperglycemia, and control of other cardiovascular risk factors. Pharmacological therapies for hepatic steatosis are limited, and lifestyle modifications follow treatment. Bariatric surgery has achieved a reduction in steatohepatitis by 84% and fibrosis by 70.2%. 7 The authors recommend adding pharmacological treatment (pioglitazone, liraglutide, obethicolic acid, orlistat), particularly in patients with advanced disease or at risk of progression 8.

Physicians usually recommend a healthy diet with less sugar (such as sugary drinks), trans fat, and saturated fat. One can substitute such a diet with polyunsaturated fats from plant oil and more omega 3 fatty acids from fish. Excessive intake of fats and carbohydrates is a risk factor for NAFLD. An unbalanced diet is strongly associated with NAFLD. It is essential to exercise regularly and lose weight. Other recommendations include not taking medicines and avoiding alcohol consumption. The liver begins to be scarring when NAFLD progresses to NASH. Fibrosis or scar tissue development should be controlled to prevent cirrhosis complications, enclosing liver failure, or cancer 4. Fat build-ups or tissue scars in the liver can be stopped or slowed down to prevent disease. Experimental treatments include antioxidants, such as vitamins E and D, along with selenium. Such agents tend to reduce the oxidative stress that builds up inside a person's liver with NASH. However, their ability to treat the disease has not yet been proven. Clinical studies have examined whether diabetes medicines can assist in the treatment of NASH. The majority of individuals with NASH are insulin sensitive, which means that insulin is incapable of effectively controlling their fatty acid levels and blood sugar in their bloodstream. Some diabetes drugs include pioglitazone, rosiglitazone, or metformin, which may aid the body to become more insulin sensitive and prevent liver damage in individuals with NASH 8. These modalities show variable results, with greater or lesser adverse effects, costs, and risks. Currently, there is no definitive therapy for these patients; future research and possible combination therapies are needed to achieve the histological benefits with minimal adverse effects 9.

Until now, the specific causes of NASH have not been clarified, and it is considered not the same in most patients. Therefore, this review summarises our current understanding, characteristics, and prevalence of NASH in diabetic individuals, mainly focusing on epidemiology, risk factors, and ethnic differences.

## Methods

### Search strategy

A systematic review of previous evidence was performed using the PubMed, Scopus, and Google Scholar databases. “Preferred Reporting Items for Systematic Reviews and Metanalyses” (PRISMA) guidelines were used to evaluate and systematically review the existing literature on the findings regarding the characteristics and prevalence of patients with diabetes with AND without NASH. The search for this systematic review was conducted between February and April 2021. While conducting a detailed and up-to-date search, complete references and citations (where available) relevant to the topic were downloaded into Endnote X9 (referencing program) for further assessment.

### Selection criteria and electronic database search

A search was conducted, and the eligibility of relevant research articles was assessed. All articles published until 2021 were scrutinised. Medical Subject Heading (MeSH) terms were identified, including “NAFLD,” “non-alcoholic fatty liver disease,” “NASH,” “non-alcoholic steatohepatitis,” “diabetes type 2,” “diabetes mellitus,” “diabetes and liver,” “fatty liver,” “hepatic insulin resistance/ diabetes,” “cirrhosis”; “fibrosis,” “prevalence,” “statosis,” and “Ethnicity.” Further articles were chosen from the search results if they were relevant to the topic of “non-alcoholic fatty liver disease or diabetes.” Followed by combining these terms using “AND,” “OR,” and also used truncation (*) mark with all search terms to retrieve all potential variants.

### Data extraction

**Figure 1:**
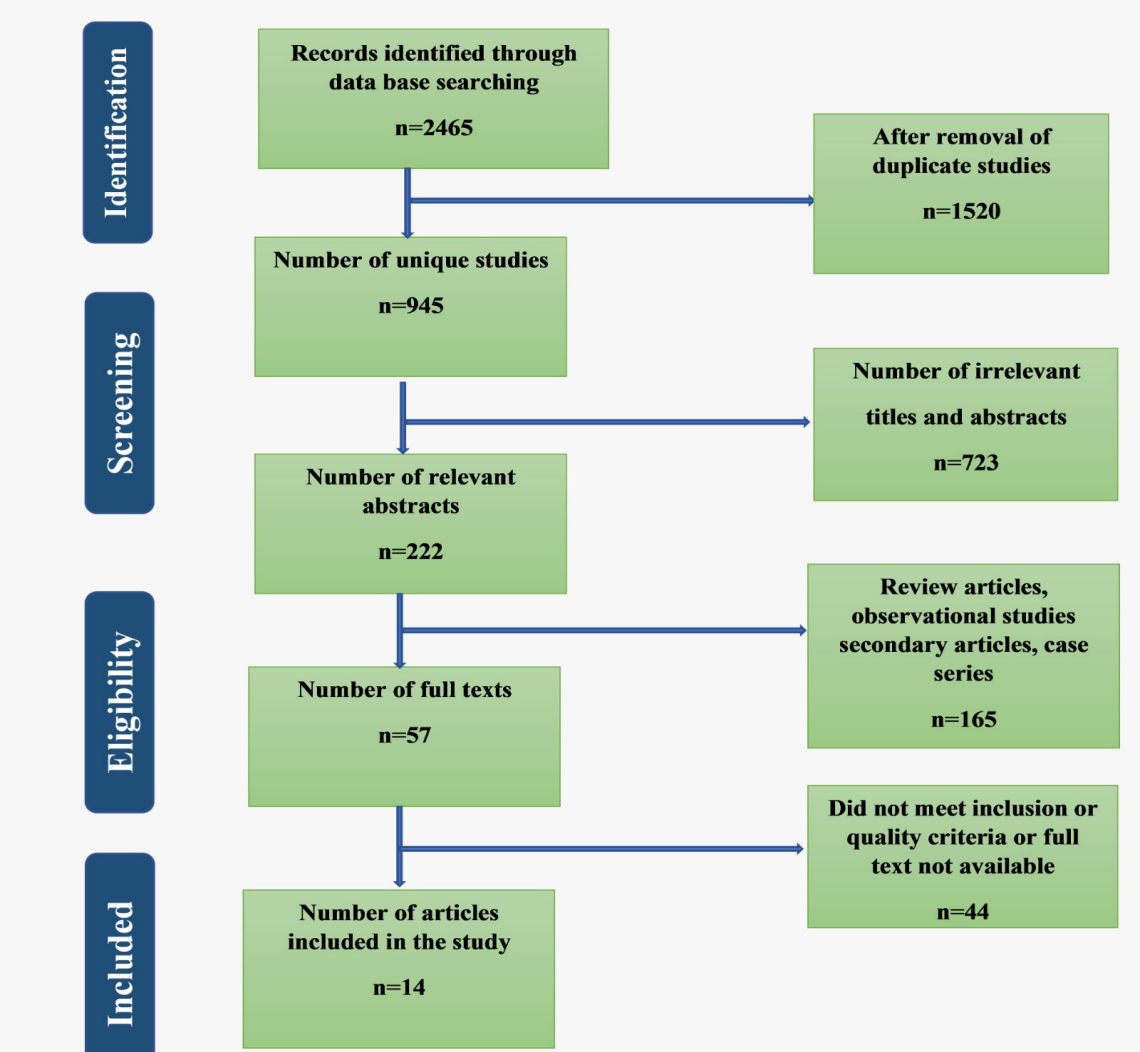
PRISMA Flow chart summarizing the selection of articles for systematic review

Studies on humans in both developed and developing countries and published in English were included in this review. All eligible primary and original studies were examined thoroughly. The snowball sampling technique was used to identify eligible articles. At the same time, references from all the scrutinised studies were reviewed to avoid any missing articles. After the identification and screening process, the eligibility of the studies was re-evaluated using the above criteria. At the same time, all qualitative studies, editorials, opinions, and without full text were excluded. The titles and abstracts of relevant articles were reviewed, and duplicates were removed. Figure 1 shows the PRISMA flowchart of the selection and screening process of the recruited studies.

## Results

### Findings of the search strategy

The articles were first searched by titles, then by abstract, and full-text articles were assessed according to the study's objectives. Initially, 2465 articles were identified through different databases; 1520 articles were found as duplicates. The remaining unique articles were assessed for eligibility criteria and the availability of the full text. Of the total, 13 articles met the objective of the systematic review and were included in the study.

### Studies Characteristics

Regarding the study design, 14 of the studies were cross-sectional, while two study were prospective or longitudinal. The sample size of all included published articles ranged from 125 to 1249 in different studies. The studies were conducted in different countries. Four studies were conducted in the USA, two in Brazil, two in Romania, two in India, one in the United Kingdom, one in Pakistan, one in Italy and one in Saudi Arabia (Table 1). Three studies were performed from 2004 to 2009, five between and 2011-2014, two in 2015, and three between 2018 to 2022, as shown in Table 1. The study participants were adults aged between 18-80years. Most studies used ultrasound-guided biopsy for the diagnosis of NASH, along with laboratory assessments.

**Table 1:** Characteristics of the included studies and the summary of main findings (n=14)

Author Year Country	Study Design/type	Study population, Sample size & age	Diagnostic criteria	Prevalence	Other findings
**Anthropometric Characteristics**					
Portillo-Sanchez, Bril (14) 2015 USA	Cross-sectional Metabolic study	103 patients with T2DM and normal plasma aminotransferase level with age 60±8years	Ultrasound-guided biopsy in patient with NAFLD by H-MRS and high liver triglyceride content (>5.5%).	NAFLD 50%. NASH 56%	Prevalence of NAFLD increase with increasing BMI (p-value 0.001).
Prashanth, Ganesh (19) 2009 India	cross sectional study	204 T2DM patient. Age 20-70 years	Laboratory analysis and Liver biopsy	87% NAFLD, 62.6% NASH 3.3% Fibrosis.	Factors that predict presence or severity of NAFLD or NASH include age, duration of DM, waist circumference, HbA1c, BMI and family history of diabetes.
Leite, Salles (28) 2009 Brazil	Cross sectional	180 T2DM patients Age > 65years	Clinical and laboratory evaluation, abdominal ultrasound	NAFLD 69.4%. Slight NASH 32%, Moderate 50.4%, Severe NASH 11.2%	High BMI, and waist circumference, were associated with NAFLD and steatosis. Multivariate analysis showed, The presence of obesity (OR: 7.1, 95% CI: 3.017.0) or Higher waist circumference (OR: 4.8, 95% CI: 1.9-12.2).
Sima, Timar (18) 2014 Romania	Cross-sectional study	348 T2DM patient with age 18-65 years	Clinical examination, blood test and abdominal ultrasonography.	NASH 87.1%,	Patient with NASH had increased abdominal circumference and BMI (p=0.001).
Alsabaani, Mahfouz (29) 2018 KSA	Cross-sectional	245 T2DM patients from primary healthcare centers	Laboratory investigation, and ultrasound	NASH 72.8%	Risk factors includes, Overweight T2DM patients (aOR = 6.112, 95% CI: 1.529-4.432), Obese (aOR = 10.455, 95% CI:2.645–41.326), Moderate diet-compliant patients (aOR = 2.413, 95% CI: 1.003-5.805) Poor diet-compliant patients (aOR = 6.562, 95% CI: 2.056-20.967).
Loomba, Abraham (30) 2012 USA	Cross-sectional	1069 patients with T2DM or have family history of DM, Age >18 years,		59.4% in patients with family history of DM and 53.4% in no family history of DM.	DM (aOR 1.76, 95% CI, 1.132.72; p-value <0.001) and family history of DM [ aOR 1.34, 95% CI 0.99-1.81, P=0.06) were associated with higher prevalence of NASH after the adjusted for age , sex, BMI, ethnicity and metabolic traits.
Williams, Stengel (31) 2011 USA	Cross-sectional study	400 patients of 18-70 years age without any liver disease attending outpatient clinics	Baseline Ultrasound; Laboratory tests; Liver biopsy	NASH 22% NAFLD 74%	Diabetes prevalence was 16.5%. Among 54 diabetic patients the prevalence of NASH was 22% with a p < 0.00005. The prevalence of NASH is higher in diabetic than non-diabetics (10.9%) Characteristics of diabetic patient with NASH include obesity (P<0.0005) and hypertension(P <0.00005)
Erminia, Lembo (32) 2022 Italy	Prospective cohort study	412 participants Age 19-67 years	Fine needle biopsy NAFLD Fibrosis Score (NFS), AST/ALT ratio, AST to Platelet ratio (APRI), fibrosis-4 score (FIB4) were calculated	NASH 63%	NASH risk double in patients with T2DM. “The prevalence of NAFL increased with the increase of BMI, while there was an inverse correlation between BMI and NASH (r=-0.145 p=0.003).”
**Laboratory Characteristics**					
Portillo-Sanchez, Bril (14) 2015 USA	Cross-sectional Metabolic study	103 patients with T2DM and normal plasma aminotransferase level with age 60±8years	Ultrasound-guided biopsy in patient with NAFLD by H-MRS and high liver triglyceride content (>5.5%).	NAFLD 50%. NASH 56%	Higher HbA1c associated with increased NAFLD prevalence and increased liver triglyceride. NAFLD Prevalence was higher in T2DM patient with normal liver aminotransferase level and 56% of them had NASH
Bazick, Donithan (33) 2015 USA	Cross-sectional	1249 patients with biopsy-proven NAFLD. Age 52.5 ± 103	Laboratory analysis and Liver biopsy	NASH 69.2% Advanced fibrosis 41.0%	Out of the total, 34.8% had diabetes. With the AUROC 0.80 ( 95% CI 0.75-0.84, p-value 0.007), the predicted model for the diagnosis of NASH in this study include white race, increased BMI, elevated AST or ALT, ferritin, increased HbA1c, albumin, waist circumference, and HOMA of insulin resistance. The above model correctly classified 67% of a diabetic patient with NASH.
Prashanth, Ganesh (19) 2009 India	cross sectional study	204 T2DM patient. Age 20-70 years	Laboratory analysis and Liver biopsy	87% NAFLD, 62.6% NASH 3.3% Fibrosis.	ALT and AST (within normal range) were significantly elevated in NASH. Increased prevalence of NASH was associated with increase in components of metabolic syndrome
Gupte, Amarapurkar (34) 2004 India	cross sectional study	100 with T2DM	Laboratory analysis and liver biopsy	12.5% NASH, 21.8% fibrosis	Mild NASH 65.5%, Moderate NASH 12.5% and severe 9.35%. No significant differences found in BMI, ALT/AST levels, serum cholesterol, and triglycerides levels.
Leite, Salles (28) 2009 Brazil	Cross sectional	180 T2DM patients Age > 65years	Clinical and laboratory evaluation, abdominal ultrasound	NAFLD 69.4%. Slight NASH 32%, Moderate 50.4%, Severe NASH 11.2%	Increased ALT and serum triglyceride were associated with NAFLD and steatosis. HbA1c and diabetic degenerative complication were not associated with NASH. Multivariate analysis showed, Increased serum triglycerides level (42.82 mmol/L, OR 3.7–4.1, 95% CI 1.2-13.3) and High-normal ALT level (Z40 U/L, OR 2.5-2.7, 95% CI 1.2-5.9) were independently associated with NASH,
Sima, Timar (18) 2014 Romania	Cross-sectional study	348 T2DM patient with age 18-65 years	Clinical examination, blood test and abdominal ultrasonography.	NASH 87.1%,	HbA1c (p<0.0001), triglyceride (p<0.001), and ALT (p=0.001) levels. 23.9% patients have elevated liver enzymes
Leite, Villela-Nogueira (12) 2011 Brazil	Cross-sectional study	125 T2DM from primary healthcare centers	Laboratory investigation, ultrasound and liver biopsy	NASH 78%, Advanced Fibrosis 34-60%,	NASH independent correlates include, Hypertriglyceridemia (p 0.034) High ALT (P 0.044) Low serum high density lipoprotein-cholesterol (P0.079). Fibrosis independent correlates include, high serum γ-glutamyl transferase (P=0.002), Older age (P=0.022) and Male gender (P=0.064).
Williamson, Price (35) 2011 UK	Prospective cohort study	939 T2DM patient with age 61-76years	Ultrasound and magnetic resonance spectroscopy	NASH 56.9%, NAFLD 42.6%	Independent predictor of NASH included higher BMI, HbA1c, triglyceride, short history of DM, low level of HDL and metformin use
Alsabaani, Mahfouz (29) 2018 KSA	Cross-sectional	245 T2DM patients from primary healthcare centers	Laboratory investigation, and ultrasound	NASH 72.8%	Risk factors includes, High ALT of more than 12 IU/L (aOR = 2.335, 95% CI: 1.096-5.062), Low HDL (high density cholesterol) (aOR = 0.044, 95% CI: 0.0050.365).
**Ultrasound findings**					
Sima, Sporea (20) 2018 Romania	Cross sectional	190 T2DM	Clinical and laboratory analysis, Transient Elastography, controlled attenuation parameter.	93.8% has NASH and 70.8% had severe NASH	Severe Steatosis was more commonly found in females (75.7%) as compared to males (p< 0.0001). 47.2 had liver fibrosis and 19.8% had cirrhosis. Mostly patients were obese, had triglycerides/HDLc ratio >4, which was correlated with NASH (p=0.04), and it was more common in severe fibrosis or cirrhosis (58.3%) than with mild or no fibrosis (36.2%)
Liaqat, Fatima (21) 2020 Pakistan	Cross-sectional	181 TDM patients with age 40-80 years	Ultrasound	57.5% with hepatostea-tois	Fatty changes were more common in obese than non-obese (p<0.0001). NASH was more common in diabetic females than males (p=0.02)
Leite, Villela-Nogueira (12) 2011 Brazil	Cross-sectional study	125 T2DM from primary healthcare centers	Laboratory investigation, ultrasound and liver biopsy	NASH 78%, Advanced Fibrosis 34-60%,	Diabetes related characteristics was not associated with NASH or fibrosis
Erminia, Lembo (32) 2022 Italy	Prospective cohort study	412 participants Age 19-67 years	Fine needle biopsy NAFLD Fibrosis Score (NFS), AST/ALT ratio, AST to Platelet ratio (APRI), fibrosis-4 score (FIB4) were calculated	NASH 63%	Only mild liver fibrosis was observed. HOMA-IR was positively associated with hepatocyte ballooning (r=0.208, p<0.0001) and fibrosis (r=0.159, p=0.008).” “The NNA highlighted a specificity of 77.3% using HDL-cholesterol, BMI, and HOMA-IR as main determinants of NASH.”

NAFLD: non-alcoholic fatty liver disease; NASH: non-alcoholic steatohepatitis; T2DM: type 2 diabetes mellitus; ALT: alanine aminotransferase; AST : aspartate aminotransferase; BMI: body mass index; FNS: NAFLD Fibrosis Score; HbA1c: haemoglobin A1C; HDL: high density lipoprotein; HOMA-IR: homeostasis model assessment-estimated insulin resistance

### Main findings

The prevalence of NASH ranged from 12% to 93.8%, the highest percentage was found in studies in Romania, and lowest in studies in India. Regarding the diagnostic criteria, ultrasound-guided biopsy of the liver remains the gold standard in most studies. However, some studies also assessed triglyceride levels (>5.5) and used MRI. Factors associated with NASH include BMI, waist circumference, glycemic index, HbA1c, and serum cholesterol levels. Other associated factors include increased age, being female, low HDL, metformin use, increased ferritin, increased albumin, and race (white).

Studies by Portillo-Sanchez et al., Bazick et al., Prashanth et al., Leite et al., Sima et al., Sima et al., Liaqat et al., Leite et al., Alsabaani et al., Loomba et al., and Williams et al. found that patients with diabetes with increased BMI were significantly associated with NASH. Similarly, some studies by (Portillo-Sanchez et al., Bazick et al., Prashanth et al., Leite et al., Williamson et al., Sima et al., Alsabaani et al.) found that high HbA1c values were associated with NASH. However, Gupte et al. and Leite et al. reported no difference with respect to diabetes-related complications in patients with NASH.

Some studies (Bazick et al., Leite et al., Sima et al., Leite et al., Williamson et al., Sima et al., Loomba et al.) reported that elevated ALT levels were found in patients with diabetes and NASH; however, two studies (Portillo-Sanchez et al., Prashanth et al.,) normal ALT levels in patients with diabetes and NASH. Portillo-Sanchez et al., Leite et al., Sima et al., Leite et al., Williamson et al., Sima et al., and Alsabaani et al. reported higher triglyceride levels in patients with diabetes and NASH.

## Discussion

NASH is a frequent disease in obese patients and/or with metabolic syndrome and has a high prevalence worldwide and nationally. Globally, 37.3% of patients with diabetes have NASH 10. However, diabetes and NASH are pathogenically interrelated 11. This can be explained by insulin resistance in addition to compensatory hyperinsulinemia leading to faulty metabolism of lipid or hepatic triglyceride (TG) build-up in NAFLD or to b-cell dysfunction in type 2 diabetes (T2DM). Previous studies suggest that the NASH-T2DM association is bidirectional! 11.

Determination of the true NASH prevalence is usually complicated; liver biopsy provides the only definitive diagnosis of NAFLD/NASH. The cost or invasiveness of a liver biopsy obscures its use as a diagnostic tool for the general population 5. If the biopsy shows scarring, fatty liver, or inflammation, NASH will be diagnosed. Liver biopsy is essential for the diagnosis of NASH and is the only technique that reliably differentiates NAFLD from NASH, despite the limitations of variability in sampling. NASH is a serious clinical condition that is often not diagnosed because liver biopsies are rarely performed for this purpose. The diagnostic tests used had low sensitivity (ALT and liver ultrasound). The fact that NASH is not diagnosed early is one reason why cryptogenic cirrhosis is much greater in patients with type 2 diabetes mellitus than in the healthy population. In this sense, researchers have pointed out that NASH is the leading cause of liver transplantation in the United States. Nevertheless, this subject is growing rapidly, so the resulting decade will witness a rapid change in the therapeutic choices available for such patients.

Prior studies in people with type 2 diabetes (or T2DM) probing this spectrum of pathology concerning NASH using liver biopsy are restricted due to limited facts and figures. However, a collective estimation of the NASH prevalence was reported to be 63%–87% in addition to moderate-severe fibrosis at 22%–60% 12. Contemporary evidence enclosing type 2 diabetic patients had a BMI of 36 kg/m2, with more than 60% of patients who underwent bariatric surgery and were known to have moderate to severe NAFLD on liver biopsy 13. The existence of diabetes is explicitly related to fibrosis in NASH. Moreover, Leite et al. 12 found that 3 out of 92 patients with type 2 diabetes had histological cirrhosis indications that were subordinate to NAFLD without clinical indication of liver disease. If validated in more extensive studies, these outcomes can form a basis for early screening of NAFLD/NASH patients with T2DM. While currently available information such as imaging techniques, biomarkers, and diagnostic panels are not sufficiently sensitive or specific, there is an increased probability that it may not take that long for them to become so in the future 14. NAFLD progression is significantly affected by lipotoxicity and insulin resistance. A study reported their lab's work already established that hepatic steatosis is correlated significantly with insulin-resistant/dysfunctional adipose tissues and not obesity as such 14.

Although studies conducted at various hepatology clinics, including patients with advanced liver diseases or cirrhosis, have pointed out the prevalence of NASH among patients with normal plasma aminotransferases 15, they left the real variable among asymptomatic patients, as they did not focus on individuals with T2DM. Another study narrowed down their included participants and reported only the prevalence among a broader otherwise healthy population with normal aminotransferases but with T2DM. This cohort is often falsely thought to be unperturbed by liver diseases, despite many of them having a cluster of NAFLD risk factors. They also examined hyperglycemia in this group of patients. Persistent exposure to elevated plasma glucose levels has long been known to cause toxicity and induce apoptosis, whereas diabetes is known to be associated with progressive NASH 6 16, 17.

The higher prevalence of NAFLD, along with the correlation of plasma HbA1c with liver triglycerides, are the key findings that indicate that hyperglycemia may play a major role in the development of hepatic steatosis and necroinflammation. Nonetheless, there are a number of other mechanisms which may play a role, such as insulin resistance/hyperinsulinemia, chronic inflammation, oxidative stress, and hepatotoxic cytokines 16, 17. Patients with T2DM often present with hepatic steatosis with more difficult-to-control hyperglycemia, worse insulin resistance, and a need for larger insulin doses 15, 16, 18. Thus, the necessity of prospective studies on patients with T2DM and NAFLD to investigate the role of reversing hyperglycemia and glucotoxicity on hepatic steatosis, necroinflammation, and fibrosis is quite evident. It is well known that there may exist a whole spectrum of histological findings of fatty liver and NASH without elevated transaminases 19.

Among adults, females with diabetes were most frequently diagnosed with NASH 20, 21. Nevertheless, this likely signifies a bias that females are more likely to go to the healthcare professionals than males (henceforth, get diagnosed with NAFLD/NASH). It is also traditionally well known that men drink more alcohol compared to women. Due to this reason, males get excluded from a diagnosis which is based on the intake of alcohol. Increasing age is also supposed to be associated with an increase in the NASH prevalence among diabetic patients 12. It is noteworthy that old people significantly suffer from more NAFLD risk factors for example, hyperlipidaemia, diabetes, obesity and hypertension 22.

Some studies have discussed the effects of various antidiabetic medication in a well characterized group of patients with poorly controlled NAFLD or Type 2 diabetes. Patients HbA1c levels, and body weight reduce by the use of metformin along with liraglutide and sitagliptin. Intrahepatic fat also declined from baseline levels in these patients 23. The insulin resistance with hyperinsulinemia is usually deleterious for the liver, and exogenous insulin in patients with type 2 Diabetes Mellitus is often worthwhile. Since NAFLD is closely connected with the insulin resistance, there comes an increased need for insulin that may have a potential for weight gain. Patients with Type 2 diabetes Mellitus may inadequately control on oral antidiabetic drugs when they are put on twelve weeks of insulin glargine therapy having hepatic fat reduction on MRS by 12.6 % to 9.9% with an improvement in HbA1c from 7.9% to 7.2%. Hence, across all the NAFLD stages during Type 2 diabetes, insulin is useful for optimizing glycemic control 24.

Metabolic syndrome has been studied as a risk factor for years, without much attention being paid to the liver. It is now well established that NAFLD and metabolic syndrome coexist frequently due to common pathophysiology. It is also well known that NASH, cirrhosis, and hepatocellular carcinoma are more common in patients with type-2 diabetes and metabolic syndrome than in those without diabetes. As a result, NAFL could be considered a crucial factor in the pathogenesis of metabolic syndrome/type-2 diabetes, cirrhosis, NASH, or even HCC complications arising from obesity/type-2 diabetes/metabolic syndrome. Therefore, patients with type-2 diabetes/metabolic syndrome and steatosis should be referred to hepatologists more frequently than they have been in the past. However, the cost-effectiveness of such frequent referrals requires close examination.

## Conclusion

NASH and NAFLD are emerging and serious public health issues that are closely associated with the continuing epidemic of type 2 diabetes. NASH is defined as the presence of stenosis and inflammation with hepatocyte damage, with or without fibrosis. Our study also provides important data characterising NASH ethnic disparities and other sociodemographic differences. As the prevalence of both NASH and type 2 diabetes increases, NAFLD/NASH management and risk stratification of NAFLD/NASH tends to pose a major challenge in patients with type 2 diabetes. It also represents a disease spectrum from simple infiltration of hepatic fat to cirrhosis and end-stage liver disease. Individuals with severe or multiple features of metabolic syndrome are at a higher risk of progressive liver disease. People at higher risk can be non-invasively investigated for the presence of significant fibrosis using liver fibrosis markers. In the absence of significant fibrosis, NAFLD patients should be managed by considering cardiovascular risk modification and lifestyle optimisation. There is a lot to learn about the disease mechanism, progression of risk factors, and targets for potential therapeutic interventions. During drug discovery, comorbidity should be taken into account with both pharmacological and pharmacogenomic considerations given to the populations identified as high-risk.

There is a scarcity of long-term studies related to the prevalence and characteristics of patients with diabetes with or without NASH. However, it is well documented that in patients with NASH, liver-related mortality is much higher than in non-NASH diseases of fatty liver 25, 26. It is also problematic, as NASH in patients with diabetes causes a higher mortality risk 27. Therefore, this study provides a solid indication for all patients with diabetes to visit a hepatologist and screen for fatty liver disease. If steatosis is found on baseline ultrasound, a liver biopsy should be performed for timely management.

## Declarations

### Availability of data and materials

The dataset generated and analysed to support this study's findings were freely available online.

## References

[1] Younossi Z, Anstee QM, Marietti M, Hardy T, Henry L, Eslam M (2018). Global burden of NAFLD and NASH: trends, predictions, risk factors and prevention. Nature reviews Gastroenterology & hepatology.

[2] Anstee QM, Targher G, Day CP (2013). Progression of NAFLD to diabetes mellitus, cardiovascular disease or cirrhosis. Nature reviews Gastroenterology & hepatology.

[3] Hagström H, Nasr P, Ekstedt M, Hammar U, Stål P, Hultcrantz R (2017). Fibrosis stage but not NASH predicts mortality and time to development of severe liver disease in biopsy-proven NAFLD. Journal of hepatology.

[4] Ahmed A, Wong RJ, Harrison SA (2015). Non-alcoholic Fatty Liver Disease Review: Diagnosis, Treatment, and Outcomes. Clin Gastroenterol Hepatol.

[5] Yki-Järvinen H (2016). Diagnosis of non-alcoholic fatty liver disease (NAFLD). Diabetologia.

[6] Wong VW-S, Adams LA, de Lédinghen V, Wong GL-H, Sookoian S (2018). Non-invasive biomarkers in NAFLD and NASH—current progress and future promise. Nature reviews Gastroenterology & hepatology.

[7] Lassailly G, Caiazzo R, Ntandja-Wandji L-C, Gnemmi V, Baud G, Verkindt H (2020). Bariatric Surgery Provides Long-term Resolution of Non-alcoholic Steatohepatitis and Regression of Fibrosis. Gastroenterology.

[8] Cernea S, Cahn A, Raz I (2017). Pharmacological management of non-alcoholic fatty liver disease in type 2 diabetes. Expert review of clinical pharmacology.

[9] Said A, Akhter A (2017). Meta-analysis of randomized controlled trials of pharmacologic agents in non-alcoholic steatohepatitis. Annals of hepatology.

[10] Younossi ZM, Golabi P, de Avila L, Paik JM, Srishord M, Fukui N (2019). The global epidemiology of NAFLD and NASH in patients with type 2 diabetes: a systematic review and meta-analysis. Journal of hepatology.

[11] Williams KH, Shackel NA, Gorrell MD, McLennan SV, Twigg SM (2013). Diabetes and nonalcoholic fatty liver disease: a pathogenic duo. Endocrine reviews.

[12] Leite NC, Villela-Nogueira CA, Pannain VLN, Bottino AC, Rezende GFM, Cardoso CRL (2011). Histopathological stages of non-alcoholic fatty liver disease in type 2 diabetes: prevalences and correlated factors. Liver International.

[13] Schauer PR, Kashyap SR, Wolski K, Brethauer SA, Kirwan JP, Pothier CE (2012). Bariatric surgery versus intensive medical therapy in obese patients with diabetes. New England Journal of Medicine.

[14] Portillo-Sanchez P, Bril F, Maximos M, Lomonaco R, Biernacki D, Orsak B (2015). High prevalence of non-alcoholic fatty liver disease in patients with type 2 diabetes mellitus and normal plasma aminotransferase levels. The Journal of Clinical Endocrinology & Metabolism.

[15] Fracanzani AL, Valenti L, Bugianesi E, Andreoletti M, Colli A, Vanni E (2008). Risk of severe liver disease in non-alcoholic fatty liver disease with normal aminotransferase levels: a role for insulin resistance and diabetes. Hepatology (Baltimore, Md).

[16] Portillo P, Yavuz S, Bril F, Cusi K (2014). Role of insulin resistance and diabetes in the pathogenesis and treatment of non-alcoholic fatty liver disease. Current Hepatology Reports.

[17] Cusi K (2012). Role of obesity and lipotoxicity in the development of non-alcoholic steatohepatitis: pathophysiology and clinical implications. Gastroenterology.

[18] Sima A, Timar R, Vlad A, Timar B, Rosu M, Dan I (2014). Non-alcoholic fatty liver disease: a frequent condition in type 2 diabetic patients. Wiener klinische Wochenschrift.

[19] Prashanth M, Ganesh HK, Vima MV, John M, Bandgar T, Joshi SR (2009). Prevalence of non-alcoholic fatty liver disease in patients with type 2 diabetes mellitus. The Journal of the Association of Physicians of India.

[20] Sima A, Sporea I, Timar R, Vlad M, Braha A, Popescu A (2018). NON-INVASIVE ASSESSMENT OF LIVER STEATOSIS AND FIBROSIS USING TRANSIENT ELASTOGRAPHY AND CONTROLLED ATTENUATION PARAMETER IN TYPE 2 DIABETES PATIENTS. Acta Endocrinologica (Bucharest).

[21] Liaqat M, Fatima M, Malik SS, Gillani SA, Manzoor I (2020). Ultrasonographic features associated with diffuse hepatosteatosis among diabetic obese and normal body mass index patients. Journal of Medical Ultrasound.

[22] Vernon G, Baranova A, Younossi ZM (2011). Systematic review: the epidemiology and natural history of non-alcoholic fatty liver disease and non-alcoholic steatohepatitis in adults. Alimentary pharmacology & therapeutics.

[23] Yan J, Yao B, Kuang H, Yang X, Huang Q, Hong T (2019). Liraglutide, sitagliptin, and insulin glargine added to metformin: the effect on body weight and intrahepatic lipid in patients with type 2 diabetes mellitus and non-alcoholic fatty liver disease. Hepatology.

[24] Tang A, Rabasa-Lhoret R, Castel H, Wartelle-Bladou C, Gilbert G, Massicotte-Tisluck K (2015). Effects of insulin glargine and liraglutide therapy on liver fat as measured by magnetic resonance in patients with type 2 diabetes: a randomized trial. Diabetes Care.

[25] Söderberg C, Stål P, Askling J, Glaumann H, Lindberg G, Marmur J (2010). Decreased survival of subjects with elevated liver function tests during a 28-year follow-up. Hepatology.

[26] Musso G, Gambino R, Cassader M, Pagano G (2011). Meta-analysis: natural history of non-alcoholic fatty liver disease (NAFLD) and diagnostic accuracy of non-invasive tests for liver disease severity. Annals of medicine.

[27] Adams LA, Harmsen S, Sauver JLS, Charatcharoenwitthaya P, Enders FB, Therneau T (2010). Non-alcoholic fatty liver disease increases risk of death among patients with diabetes: a community-based cohort study. The American journal of gastroenterology.

[28] Leite NC, Salles GF, Araujo ALE, Villela-Nogueira CA, Cardoso CRL (2009). Prevalence and associated factors of non-alcoholic fatty liver disease in patients with type-2 diabetes mellitus. Liver international.

[29] Alsabaani AA, Mahfouz AA, Awadalla NJ, Musa MJ, Al Humayed SM (2018). Non-alcoholic fatty liver disease among type-2 diabetes mellitus patients in Abha City, South Western Saudi Arabia. International journal of environmental research and public health.

[30] Loomba R, Abraham M, Unalp A, Wilson L, Lavine J, Doo E (2012). Association between diabetes, family history of diabetes, and risk of non-alcoholic steatohepatitis and fibrosis. Hepatology.

[31] Williams CD, Stengel J, Asike MI, Torres DM, Shaw J, Contreras M (2011). Prevalence of non-alcoholic fatty liver disease and non-alcoholic steatohepatitis among a largely middle-aged population utilizing ultrasound and liver biopsy: a prospective study. Gastroenterology.

[32] Lembo E, Russo MF, Verrastro O, Anello D, Angelini G, Iaconelli A (2022). Prevalence and predictors of non-alcoholic steatohepatitis in subjects with morbid obesity and with or without type 2 diabetes. Diabetes & Metabolism.

[33] Bazick J, Donithan M, Neuschwander-Tetri BA, Kleiner D, Brunt EM, Wilson L (2015). Clinical model for NASH and advanced fibrosis in adult patients with diabetes and NAFLD: guidelines for referral in NAFLD. Diabetes care.

[34] Gupte P, Amarapurkar D, Agal S, Baijal R, Kulshrestha P, Pramanik S (2004). Non-alcoholic steatohepatitis in type 2 diabetes mellitus. Journal of gastroenterology and hepatology.

[35] Williamson RM, Price JF, Glancy S, Perry E, Nee LD, Hayes PC (2011). Prevalence of and risk factors for hepatic steatosis and non-alcoholic fatty liver disease in people with type 2 diabetes: the Edinburgh Type 2 Diabetes Study. Diabetes care.

